# Lanolin-Based Synthetic Membranes for Transdermal Permeation and Penetration Drug Delivery Assays

**DOI:** 10.3390/membranes11060444

**Published:** 2021-06-15

**Authors:** Cristina Alonso, Ilaria Collini, Meritxell Martí, Clara Barba, Luisa Coderch

**Affiliations:** Surfactants and Nanobiotechnology Department, Institute of Advanced Chemical of Catalonia of CSIC, (IQAC-CSIC), Jordi Girona 18-26, 08034 Barcelona, Spain; cristina.alonso@iqac.csic.es (C.A.); ilariacollini77@gmail.com (I.C.); meritxell.marti@iqac.csic.es (M.M.); clara.barba@iqac.csic.es (C.B.)

**Keywords:** lanolin, synthetic membranes, skin, permeation, penetration

## Abstract

Due to the high similarity in composition and structure between lanolin and human SC lipids, we will work with two models from wool wax. Two types of lanolin were evaluated: one extracted with water and surfactants (WEL) and the other extracted with organic solvents (SEL). Skin permeation and skin penetration studies were performed with two active compounds to study the feasibility of the use of lanolin-based synthetic membranes as models of mammalian skin. Diclofenac sodium and lidocaine were selected as the active compounds considering that they have different chemical natures and different lipophilicities. In the permeation assay with SEL, a better correlation was obtained with the less permeable compound diclofenac sodium. This assay suggests the feasibility of using artificial membranes with SEL as a model for percutaneous absorption studies, even though the lipophilic barrier should be improved. Penetration profiles of the APIs through the SEL and WEL membranes indicated that the two membranes diminish penetration and can be considered good membrane surrogates for skin permeability studies. However, the WEL membranes, with a pH value similar to that of the skin surface, promoted a higher degree of diminution of the permeability of the two drugs, similar to those found for the skin.

## 1. Introduction

Human skin offers an affordable and easy route of administration for many drugs. Transdermal drug delivery is particularly useful when a drug is poorly absorbed when administered orally (the most commonly used route for most drugs) or if the drug cannot tolerate the harsh surroundings of the gastrointestinal (GI) tract.

Human skin comprises the epidermis (nonvascular layer about 100 µm thick), the dermis (a highly vascularized layer about 500 to 3000 µm thick), and the underlying subcutaneous tissue, with sebaceous and sweat glands running throughout. The outermost epidermal layer is the SC or horny layer (10–40 µm thick) and represents the main barrier to skin permeation by dermally applied drug formulations. Due to the exceptional SC lipid composition, with long-chain ceramides, free fatty acids, and cholesterol as main lipid classes, the lipid phase behavior is different from that of other biological membranes [1,2]. The SC provides its greatest barrier function against hydrophilic compounds, whereas the viable epidermis is most resistant to highly lipophilic compounds [3]. In vivo experiments on humans are expensive, time-consuming, and morally undesirable. Therefore, alternatives to in vivo studies in humans have been explored. The suitability of different in vitro permeability models has been largely reviewed [4,5], using (human or animal) excised skin to mimic in vivo studies. Moreover, the main inconvenience of using biological membranes is the high intra- and inter-subject variability [6].

Additionally, attempts have been made to create synthetic membranes that can be used as human skin models to investigate the transdermal diffusion properties of pharmaceutical and cosmetic formulations [7]. Recently, great effort has been put into developing artificial membranes as surrogates for human skin [8,9]. Such synthetic membranes are made up of a thin film of polymeric macromolecules that can content the transfer of components through them. They may be made of synthetic polymers (e.g., polycarbonate, polysulphone) or semi-synthetic cellulose polymers (e.g., cellulose nitrate, cellulose acetate). However, additional attempts must be made to mimic the complex composition of the lipid structure of the SC [4].

The lanolin structure mimics the lipid matrix of SC by having similar properties and chemical composition, then it may provide a suitable strategy to provide accurate modeling of skin barrier properties by combining it with synthetic membranes. Wool wax and SC lipids have been shown to share important characteristics: cholesterol and its derivatives, certain free fatty acids, and ceramides and have been found in lanolin [10,11,12]. In addition, wool wax and SC lipids can both co-exist as liquids and solids at physiological temperatures [13,14,15]. In the present study, lanolin was extracted not only by the usual water-based process (WEL) but also by a solvent-based process (SEL) through a close loop process for scouring wool fibers designed in a so-called "wool dry scouring process” (WDS) [12]. It is important to highlight that in the WDS process, the extracted lanolin has more amount of polar lipids and therefore with higher resemblance to SC lipids from human beings [13].

The present study concerns whether the inclusion of lanolin in a synthetic membrane (Nuclepore^®^) enhances the membrane barrier and mimics mammal skin. Dermatomed porcine skin has been used in this type of research because the permeability of pigskin is similar to that of humans [16]. Nuclepore^®^ is a 10 µm thick filter in which a pore size of 0.05 nm in a 25 mm diameter polycarbonate membrane [17]. An indicator of membrane integrity/barrier functions the permeability of the membrane which is assessed by measuring transepidermal water loss (TEWL) [16].

In transdermal studies, two main in vitro systems, which yield different parameters, are widely studied to outline the transport of drugs. Penetration, which usually involves finite dosing, is possible to obtain information about the total amount of drug detectable in the skin after different incubation times and the drug concentration in different skin layers. Permeation, which usually involves infinite dosing, carriers to data that provide information about the amount of drug that has permeated through the skin after different time points, such as steady-state flux, permeability, and the diffusion constant [18].

There are many QSAR models, and they have to be applied to new data without introducing additional degrees of freedom by performing analyses such as linear regression on the predicted and experimental data. Only then can the predictive power of the model be assessed [19]. Numerous QSAR models exist to predict the permeability coefficient or flux of permeants, which are normally used to forecast the cumulative amount of drug permeated in infinite dosing [20]. In this paper, the Potts and Guy equation, which considers the solute octanol-water partition coefficient (K_o/w_) and the molecular weight (MW) was applied [21].

The transdermal behavior of two drugs with different physicochemical properties using penetration/finite and permeation/infinite dosing systems were studied in this work. The results are related to the QSPRs, normally used on chemical absorption prediction through and into h the skin. The interrelationship between permeation and skin penetration data has been sought by taking into account the ionization of the different active ingredients in the different skin layers studied. The absorption of lidocaine and diclofenac sodium, widely employed in dermatology, were studied having in mind their different molecular weights and hydrophilic/hydrophobic balances. Since the lipophilicity of a substance is a key parameter for its skin absorption, the octanol/water distribution coefficient (Log D) of the two active substances was also determined at physiological pH 7.4 and the skin surface pH 5.5. The barrier function of lanolin-containing artificial membranes has been recently studied using penetration with finite dosing methodology [22] and using a permeation with infinite dosing methodology [23].

Therefore, the main objective of this work was to determine the advantages and disadvantages of lanolin-based synthetic membranes in skin penetration and permeation and penetration studies as models of mammalian skin. Two types of lanolins were used, which slightly acidify the medium and lead to important changes in the lipophilicity of the active ingredients and consequently, their permeation and penetration. Comparison of the results obtained with the lanolin membranes and human skin will support the use of these lanolin membrane models in topical penetration and permeation assays.

## 2. Materials and Methods

### 2.1. Materials

Diclofenac sodium (DS) (log K_o/w_: 1.1, MW: 318.1), lidocaine (LIDO) (log K_o/w_: 2.4, MW: 234.3), phosphate-buffered saline (PBS), and propylene glycol (PG) were acquired at Sigma (Sigma-Aldrich, St. Louis, MO, USA). DS 3% (*w/V*) and LIDO 2% (*w/V*) in PG were tested on the permeation and penetration assays. Nuclepore^®^(NP) polycarbonate membranes were provided by Sigma (Sigma-Aldrich, St. Louis, MO, USA). HPLC-grade eluents, solvents, and buffers (sodium dihydrogen phosphate monohydrate (NaH_2_PO_4_·1H_2_O)) used to perform the experiments were supplied by Merck (Merck, Darmstadt, Germany).

Solvent extracted lanolin (SEL) was produced by Spanish Merino sheep extraction according to the Dry Wool Scouring Eco-Efficient process of the pilot plant, described in LIFE 11 ENV/ES/588 [12]. This resulted in the extraction of lipids with hexane (Merck, Darmstadt, Germany) and the elimination of impurities by centrifugation. After processing, lanolin was removed from the hexane by distillation at 35 °C. Water extracted lanolin (WEL) was obtained following a standard procedure with surfactant in an aqueous medium and was supplied by Tavares (Textil Manuel Rodrigues Tavares, Guarda, Portugal).

### 2.2. Preparation of Pig Skin Membrane

Pigskin was used as a standard for both permeation and penetration tests. The handling of the animals involved in these trials was in accordance with the Guide for the Care and Use of Laboratory Animals published by the National Institutes of Health in the United States [24]. The Animal Ethics Committee and the Institutional Review Board and the University of Barcelona, Barcelona, Spain, approved the protocol (28 January 2013). The skin was supplied by the Departmento de Cardiología, Hospital Clinic, Barcelona, Spain. Unboiled pig skin was obtained from the back area of different weaned white/landrace pigs weighing 30–40 kg after euthanization. The pig bristles were carefully removed with an animal razor and the hairless skin was rinsed with water. The skin was dermatomized with a Dermatome GA630 (Aesculap, Tuttlingen, Germany) to a thickness of 500 ± 50 μm measured with a digital micrometer (Mahr, Göttingen, Germany) and cut into 2.5 cm diameter pieces. The skin was then kept at −20 °C until used.

### 2.3. Artificial Lanolin Membrane Preparation

SEL and WEL lanolin were utilized in solutions of hexane:96% ethanol (2:1) at a concentration of 5% (*w/V*). For the preparation of the membranes, the procedure described by Pullmannová, et al. [25] was followed. Membranes were firstly hydrated in hexane:96% ethanol (2:1) and then they were dried at ambient temperature. Next, 300 µL of the lipid solution was spread onto the pre-weighed NP membranes (≈15 mg lanolin) in a three-step process. The solvent was then evaporated under a stream of N_2_. The lipid membranes prepared were vacuum-dried over P_5_O_10_ in a desiccator and were stored in a fridge at 5 °C for 24 h. Prior to use, the lanolin-based membranes were then heated to 75 °C, equilibrated for 10 min, and then cooled down slowly (~3 h) to 32 °C. The last part of the cooling-heating cycle was incubation for 24 h at 32 °C. Using a skin pHmeter (Sebumeter pH 900, Courage + Khazaka, Cologne, Germany), the pH of the membranes was determined.

### 2.4. Water Permeability by Trans-Epidermal Water Loss (TEWL)

Before starting the penetration and permeation assays, humidity, TEWL, and temperature were measured for all membranes. TEWL determination is a well-established methodology for assessing skin barrier integrity. As stated in the OECD Guidelines for the Testing of Chemicals [26], only porcine skin with a TEWL below 15 g/h-m^2^ can be used for permeability tests. This guaranteed the exclusion of holes through which the compounds tested could flow, as well as the uniformity of the skin. TEWL values were determined for skin membranes and all artificial membranes. The measurements were performed with a Tewameter TM 300 (Courage + Khazaka, Cologne, Germany).

### 2.5. Finite-Dose Penetration Test using Vertical Franz Diffusion Cells

The excised pigskin or membranes were placed in a static Franz diffusion cell (Lara Spiral, Couternon, France) with the SC side towards the donor compartment. The surface area was 1.86 cm^2^, and the capacity of the receiver chamber was around 3 mL. The receiver chamber was then filled with receptor fluid (RF); phosphate-buffered saline at pH 7.4 (Sigma-Aldrich, St. Louis, MO, USA) in Milli-Q water for DS and NaH_2_PO_4_ (0.05 M, pH = 7) for LIDO. Air bubbles were gently removed in between the skin and the RF with a syringe. Finally, the assembled Franz-type cells were transferred to a regulated temperature water bath on a magnetic stirring plate and agitated at 700 rpm to maintain homogeneity of the receptor fluid. The water bath was kept at 40 °C to achieve a membrane surface temperature of 32 ± 1 °C. Once the skins were stable in the water bath, the permeability and integrity and of all membranes were studied by determining TEWL value, as detailed above. Lidocaine and diclofenac sodium were selected to study their permeation through the skin and the different lanolin membranes. The concentrations of the active drug solutions were 2% (*w/V*) for lidocaine and 3% (*w/V*) for sodium diclofenac. All solutions were prepared in propylene glycol, and 20 µL of each PG solution (finite dose) was applied to the membrane surface. PG was chosen as a solvent because of its great solubility for the different active ingredients. However, it has to bear in mind a possible lanolin distortion as it’s known in the case of the skin [27]. The PG solutions were tested in triplicate in each membrane. The finite assay protocol was conducted as described in a previous study [22]. The quantity of activity in the samples was measured using an HPLC methodology that was validated according to the ICH Q2 (R1) guidelines in terms of accuracy, linearity, and precision [28]. The results are reported as the normalized amounts (%) of substance penetrated and their standard deviations. The quantity of drug that permeated the skin was assumed to be the sum of that in the receptor fluid, epidermis, and dermis [26].

### 2.6. Infinite-Dose Kinetic Permeation Assay using Vertical Franz Diffusion Cells

Kinetic diffusion experiments were carried out by using a Vision Microette Autosampler with an Autofill Collector (Hanson Research, Chatsworth, CA, USA) in automated vertical diffusion cells. The exchange surface area between the chambers was 1.77 cm2 and the receiving chamber had a volume of about 7 mL. Similar to the penetration assay, the concentrations of the solutions of drug active compounds were 2% (*w/V*) for lidocaine and 3% (*w/V*) for diclofenac sodium in propylene glycol.

The receptor fluid (RF) employed was an aqueous buffer solution (pH 7.4) for DS and NaH_2_PO_4_ (0.05 M, pH = 7) in the case of LIDO. Once the recipient chamber was loaded with RF, forming a meniscus, the membranes were put in place, then the donor chamber. Lastly, the entire system was clamped shut. Due to the use of a propeller mixer and a magnetic stirrer (700 rpm), the recipient solution remained well mixing. The water bath was placed at 40 °C to achieve a membrane surface temperature of 32 ± 1 °C.

After measuring the TEWL, 300 μL (infinite dose) of the two solutions were applied to each Franz cell in triplicate. At different times of sampling (15, 30 min, 1, 2, 4, 6, 10, 20, and 30 h), the autosampler picked up 0.7 mL aliquots from each cell, which were then transferred and analyzed as previously described [21]. The active pharmaceutical ingredient (API) release was assessed by the cumulative amount released (*Qn*, μg/cm^2^) [29]. The equation for this determination is as shown in Equation (1):

(1) $$Qn=\frac{Cn\times Vc+\sum_{i=1}^{n-1}\left(Ci\times Vs\right)}{A}$$

where *Qn* is the cumulative amount of active ingredient delivered at time n (μg/cm^2^); *Cn* is the active ingredient concentration in the sample (μg/mL); *Vc* is the vertical diffusion cell volume (7 mL); $\sum_{i=1}^{n-1}\;Ci$ is the sum of the API concentrations (µg/mL) determined at intervals 1 to n-1; *Vs* is the volume of the sample; *A* is the sample surface area (1.77 cm^2^). The cumulative percent API permeated over time was estimated using Equation (2):

(2) $$\%\;Released\;API=\frac{Qn}{Total\;amount\;of\;API\;applied}\times 100$$

*Qn* and % API released, were both used to produce graphs displaying the absorption and penetration kinetics on the Y-axis. Time or its square root (*√t*) is indicated on the X-axis. The released API percentage overtime was adjusted to the four Equations (3)–(6), to represent the adsorption kinetics [30].

(3) $$\mathrm{Zero}-\mathrm{order}:\;\frac{\%Rt}{\%R\infty }=K_{0}\times t$$

(4) $$\mathrm{First}\;\mathrm{order}:\;\frac{\%Rt}{\%R\infty }=1-e^{k\cdot t}$$

(5) $$\mathrm{Higuchi}:\;\frac{\%Rt}{\%R\infty }=kh\times t^{1/2}$$

(6) $$\mathrm{Korsmeyer}–\mathrm{Peppas}:\;\frac{\%Rt}{\%R\infty }=K\times t^{n}$$

This step enabled us to determine the best adsorption model to support the API kinetics penetration across the membranes. The fitting of each model was determined by the non-linear regression software STATGRAPHICS Plus 5 (Statgraphics Technologies, Inc., The Plains, VA, USA), and the optimal equation was chosen according to the highest correlation coefficient adjusted for the number of degrees of freedom (R^2^ DoF). After defining the model, it was then possible to estimate the following parameters: flux (*J*), delay time, maximum time (*t max*), permeability coefficient (*Kp*), maximum concentration (*C max),* and area under the curve (AUC).

### 2.7. High-Pressure Liquid Chromatography-Diode Array Detector (HPLC-DAD) Analysis

Analyses were conducted by reversed-phase HPLC using a HITACHI ELITE LaChrom VWR instrument (Labexchange, Burladingen, Germany) with a piston pump (HTA L-2130), an autosampler (L-2200), and a diode-array detector (DAD-detector 5430). The software package was EZChrom Elite c 3.1.6, (Agilent Technologies, Santa Clara, CA, USA).

The validation of the analytical methods complied with the guidelines defined by the International Conference on Harmonisation (ICH) [28]. The ICH guidelines were followed to establish the limit of detection (LoD), the limit of quantification (LoQ), and the calibration curve. Intra- and inter-day precision of the method was determined. The analytical conditions of the HPLC-DAD and isocratic method for the two active ingredients are listed in Table 1. These conditions were the same for the starting solutions and the analyses aliquots of the kinetic permeation and Franz cell penetration tests.

### 2.8. Statistical Analysis

Statistical analysis was performed with the non-linear regression software STAT-GRAPHICS Plus 5 (Statgraphics Technologies Inc., Virginia, USA).

The Kruskal-Wallis test is a non-parametric test that is used in the case of the non-normal statistical distribution of the data. It was employed to match the permeability parameters of the different membranes with the porcine skin values. Significance was checked at the 0.05 level of probability (*p*). All results are reported as the mean ± standard deviation (SD).

## 3. Results and Discussion

### 3.1. Evaluation of Lanolin-Based Membranes

Lanolin-based synthetic membranes were formed on Nuclepore^®^ membranes using two types of lanolin, solvent extracted lanolin (SEL) and water extracted lanolin (WEL) at a concentration of 5% as described in the experimental section. The quantity of lanolin attached to the membranes was assessed, as well as the pH values of the membrane surfaces. In addition, the membranes were placed in the donor Franz cell chamber and the recipient fluid in the receptor chamber and TEWL was determined as described above. The amount of lanolin on the membrane surface, pH, and TEWL results of the lanoline-based membranes, the skin membrane, and NP are shown in Table 2.

For the two SEL and WEL membranes, the amount of lanolin found in the membrane was very similar to that applied (15 mg), which indicates that all lanolin was properly bound, with SE lanolin being slightly more bound than WE lanolin.

The surface pH of lanolin-based membranes is a crucial parameter to determine. It can yield information on the percentage of ionization of APIs in the vicinity of the membranes, thus determining the capacity of the API to pass through. The pH values measured were approximately 6.5 for the SEL membranes and 5.5 for the WEL membranes. It is noteworthy the similar pH values of the WEL membranes and the skin.

TEWL is critical to guarantee the integrity of the skin. In the present work, water permeability was assessed to evaluate the resemblance between the artificial membranes and the skin. The TEWL value of the NP membrane was extremely high; in this case, the synthetic membrane only acts as a support and not as a barrier to avoid the evaporation of water. The skin presents a TEWL an approximate value of 6 g/h·m^2^, which is low enough to ensure that the control of the excised skin was intact. The TEWL value of the artificial lanolin-based tissue membranes diminished similarly, the addition of lipids causing the formation of a thicker barrier that prevented the evaporation of water. No statistically significant differences were found for values of TEWL for lanolin membranes compared to skin. This suggests that the lanolin-induced barrier in these particular membranes was similar to that of the skin.

### 3.2. Physico-Chemical Properties of the Active Compounds

Diclofenac sodium and lidocaine were selected as the active compounds, considering they are commonly used in topical formulations and because of their different chemical identities (acidic and basic, respectively) and different lipophilies. Their respective distribution coefficients (Log D values) at pH 5.5 and 7.4, and their molecular weights, were in silico calculated using Chem Axon (ChemAxon, Budapest, Hungary). Their different physicochemical properties allowed us to conclude the link between these active properties and the permeability predictions obtained from the in silico and in vitro models. The skin permeability coefficients (log Kp values) were calculated with the Potts and Guy model [21].

Log D was determined at two different pH values; at pH 7.4 because this is the pH of the blood and the receptor fluid and at pH 5.5 because this is the pH of the skin surface [31,32]. Lidocaine (base) and diclofenac sodium (acid) showed different behaviors. Diclofenac sodium is mostly negatively charged at pH 7.4 and has a lower Log D than at pH 5.5 when it presents an unionized fraction with a higher Log D. In contrast, lidocaine is mostly positively charged at pH 5.5 and has a lower Log D than at pH 7.4, with a greater unionized fraction and a higher Log D. These data could be important since skin penetration is favored for unionized compounds [33,34]. Therefore, higher permeation of diclofenac sodium at pH 5.5 and lidocaine at pH 7.4 would be expected. Furthermore, these compounds have different molecular weights and lipophilicity (partition coefficient in octanol/buffer (Log D)) (Table 3).

The Potts and Guy equation takes into account the solute octanol-water partition coefficient (log *K_o/w_*) and the molecular weight (*MW*) (Equation (7))

(7) $$\log K_{p}=0.711\log K_{o/w}-0.0061MW-6.3$$

This model considers active lipophilicity, as the most important parameter for skin permeation. Lidocaine is the most lipophilic compound (at neutral pH) with a smaller molecular weight; therefore, it has the highest log *K_o/w_*, which theoretically indicates greater permeability.

### 3.3. Transdermal Drug Delivery Assays

Based on the EMA [35], for the assessment of transdermal absorption, human skin is the most appropriate membrane, even though its limited availability. Many animal models have been proposed, and the most significant animal model for human skin is porcine skin [36]. Recently, alternatives for skin have been widely discussed, such as artificially cultured human skin models, a parallel artificial membrane permeability assay (PAMPA), and artificial membranes [8,37,38]. In general, the use of skin cultures is restricted due to their lack of efficacy as a barrier in skin permeability studies, as well as their reproducibility and cost. Recently, great efforts have been put into developing artificial membranes as surrogates for human skin [8]. Thus, the barrier functions of lanolin-containing artificial membranes have recently been studied [22,23], which indicates that they mimic topical active absorption. Therefore, the main aim of this work was to determine the pros and cons of lanolin-based synthetic membranes in both skin permeation and penetration studies as models of mammalian skin.

#### 3.3.1. Finite Dose Drug Delivery Assay

As described in the methodology, lidocaine and diclofenac were applied to Franz cells on pig skin membranes, Nuclepore^®^ (a lanolin-free synthetic membrane), and Nuclepore^®^-SEL (a lanolin-based synthetic membrane). SEL lanolin was chosen due to the important amount of polar lipids in its composition and hence greater human skin resemblance [13]. The amount of active ingredient in the receptor fluid after 24 h was determined for all artificial membranes and is expressed as a percentage. When porcine skin was used, the amount of active ingredients was evaluated in the stratum corneum, epidermis, dermis, and receptor fluid. Skin permeation is considered as the total quantity from receptor fluid, the epidermis, and the dermis [26].

**Figure 1:**
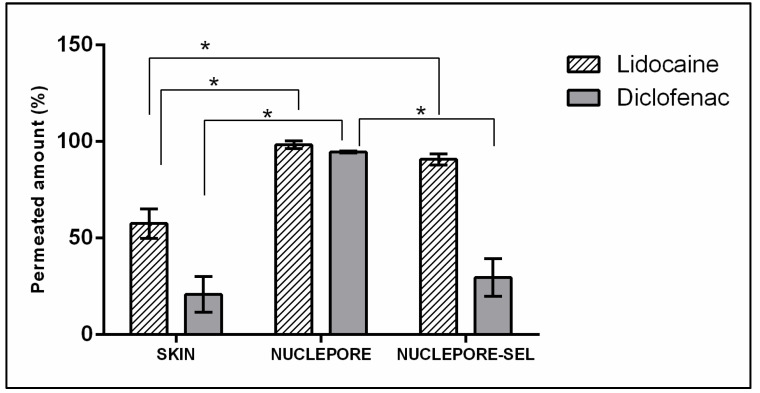
Normalized permeated amounts (%) in the skin, Nuclepore^®^, Nuclepore^®^-SEL for lidocaine, and diclofenac sodium. (*) denotes a *p*-value ≤ 0.05 from the test of Kruskal Wallis.

When the active ingredients were applied to the skin membrane, lidocaine clearly showed higher permeation than diclofenac sodium (Figure 1). The permeation results with Nuclepore^®^ confirmed the conclusions made by looking at the TEWL values. The two different active ingredients permeated much better through Nuclepore^®^ than through skin, with significant differences. However, when SEL lanolin was present in the membrane, especially in the case of diclofenac sodium, an insignificant difference compared with the skin was found.

The addition of lanolin to the Nuclepore^®^ artificial membranes resulted in a reduction of TEWL values and moderation of the permeation of the two different actives, yielding results similar to those obtained with skin. However, a better correlation was obtained with the less permeable compound diclofenac sodium, which was more hydrophilic and had a higher molecular weight than the more permeable compound lidocaine. It seems that the lanolin barrier has to be improved for highly lipophilic active compounds. Lanolin has been shown previously to reinforce SC lipids, which has led to improved skin barrier function in topical in vivo studies [13]. This assay suggests the feasibility of using artificial membranes with SEL lanolin as a model for percutaneous absorption studies, even though the lipophilic barrier should be improved.

#### 3.3.2. Infinite Dose Drug Delivery Assay

As previously noted, the lanolin-based membranes were formed with Nuclepore^®^ membranes, and the two different types of lanolin, water-extracted (WEL) and solvent-extracted (SEL), at a concentration of 5%. The solvent-extracted lanolin membranes were chosen to be assayed because of their lipid composition in the previous trial. In this case, water-extracted lanolin was also assayed because it presents both a similar diminution of the TEWL value and an acidic pH similar to that of the skin, which is fundamental for permeation studies.

**Figure 2:**
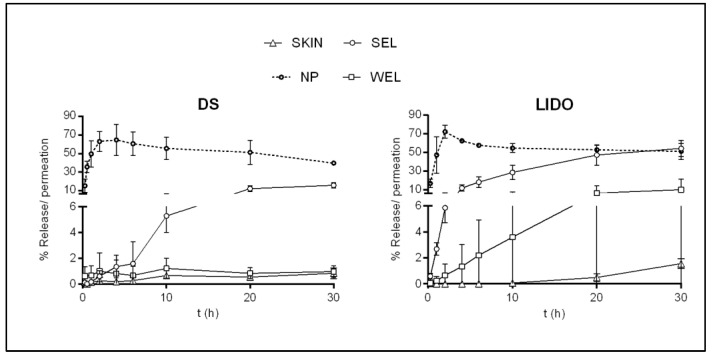
Average percentage release of lidocaine (LIDO) and diclofenac sodium (DS), through Nuclepore^®^, 5% SEL and 5% WEL membranes and average percentage permeation of these compounds through the skin.

Kinetic studies were conducted using Vision Microette automated vertical diffusion cell equipment. Kinetics was assayed for each active drug in triplicate on the skin and NP, SE, and WE lanolin membranes. The results are reported as the percentage of release (%) over time and are displayed in Figure 2.

Through the skin, the drug permeated very slowly due to the SC barrier, which made the compounds reach the RF very poorly. Lidocaine, the more lipophilic compound with the smaller MW, presented a permeability of 1.74 ± 0.53% at 30 h, while diclofenac sodium, which is more hydrophilic with a higher molecular weight, presented an even lower permeability of 0.83 ± 0.42% at 30 h. On the contrary, the NP membrane provided the highest release in both cases, as there was no barrier to the flow of the actives. The two APIs could pass through the membrane very quickly, and their highest release was achieved between 2 and 4 h.

The behavior of active drug kinetics was compared across lanolin membranes, skin membranes, and NP membranes. Maximum liberation was 30 h from the skin as well as from lanolin-based membranes. The penetration through the WEL membranes was lower in both cases than that through the SEL membranes. Similar to the finite dosage penetration assay, the DS permeation profile was more likely to that of the skin for the two membranes. However, the marked diminution of permeation when using the WEL membrane was also observed for the more lipophilic compound. WEL membranes gave results more similar to those of the skin, which could be associated with the different lipid components of the extracted wool wax. The WEL was shown to be the one richest in non-polar lipids [13]. This could promote the creation of a more robust barrier difficult to overcome for hydrophilic and lipophilic compounds such as DS and LIDO.

In summary, even with the barrier effect from the SEL membranes, the WEL membranes blocked the release to a greater extent, in a similar way to that of the skin, demonstrating the feasibility of establishing a more solid barrier for both hydrophilic and hydrophobic compounds.

Equations (3)–(6) were adjusted with the individual results for percentage release (%) over time for each membrane and each API. Once the most accurate equation describing the release was determined, the parameters of permeability coefficient (Kp), flux (J), lag time, maximum concentration (Cmax), and area under the curve (AUC) were estimated. The average results are listed in Table 4.

The skin permeation kinetics for both compounds followed a zero-order model. This model pointed to the controlled delivery of the API due to the selective membrane imposition and not to the dosage applied. The main cutaneous barrier of the skin resides in the stratum corneum lipid layer. The stratum corneum of the skin acts as a reservoir for the compounds, enabling a controlled and steady release of APIs from the SC to the viable epidermis. On the other side, the kinetic delivery from the membrane of the NP was best fitted to a Higuchi model. This model outlines passage diffusion due to a concentration gradient of the actives. In this scenario, the release was not restrained by any physical hindrance from a membrane. Due to the absence of a barrier in this membrane, the kinetic parameters were significantly higher than those of the skin.

The data from the release of the actives from the different lanolin-based membranes were also fitted to the models, different findings were observed. Only lidocaine from the SEL membranes fitted best to the Higuchi model, but from SEL membranes and LIDO and DS from the WEL membranes followed the same zero-order kinetics as the skin. Based on these data, it can be generally concluded that the presence of WE lanoline provided a barrier able to control the release of the active compound with kinetics similar to the skin. In addition, statistically similar kinetic parameters were determined for DS and LIDO using the WEL membranes. In general, it can be derived that the presence of WE lanolin as a protective barrier was the most closely resembling the skin without significant differences for either of the active compounds. However, this barrier is slightly better for hydrophilic compounds than for hydrophobic compounds.

Despite the low release of the two active ingredients through the skin, the order of penetration of the two active compounds indicated a higher lidocaine release than that of diclofenac sodium at 30 h. This works for all cases, although differences are reflected in the in silico values. Table 5 presents the Log Kp values obtained using pigskin, the lanolin membranes, and the values obtained in silico.

These data are in accordance with the highest log D observed at pH 7.4 and the lowest MW for the compound with the greatest permeation. LIDO is a base (pKa ≈8), and at a pH of 5.5 (the pH of the skin surface), it would be highly protonated; therefore, its ionized form would be absorbed slowly during the first hours. Subsequently, LIDO begins to penetrate due to its good lipophilicity and the pH gradient of the skin [39], and the receptor fluid (pH of 5.5 at the SC surface, but becoming progressively more alkaline as it moves towards the stratum granulosum) [40,41]. The acid pH value on the skin surface is due to the water-soluble substances of the stratum corneum (sebum, secreted sweat, natural moisturizing factor (NMF), and acid lipids) that are deposited on its surface. This pH gradient factor is acting as a motive force for the permeation of LIDO, whose presence in the SC acts as a sort of depot due to the ionization of the amino group. Once the inner layers of the skin are reached by LIDO, permeation is enhanced, and the absorption rate increases due to the higher pH, causing a larger proportion of the un-ionized form to more readily penetrate. DS is an acid (pKa ≈ 4) that ionizes at the skin’s surface pH, which together with its increased hydrophilicity and MW leads to delayed penetration. In addition, the increased pH inside the skin results in slower and lower absorption. The great similarity between log Kp using skin and the WEL membrane, which presents a pH of 5.3 that is analogous to that from the skin, is clear. The log Kp from the SEL membrane is in the same range with a slightly higher permeability but is much more similar to the in silico values. Therefore, the two membranes can be considered good membrane surrogates for skin permeability studies.

In previous work, solvent-extracted lanolin (SEL) and water-extracted lanolins (WELs) were analyzed, and their effectiveness as SC lipid mimics was evaluated when applied to the skin in vivo [13]. SEL was found to have a significantly higher polar lipid content enriched with compounds that degrade at higher temperatures than that of the water-extracted lanolin. Furthermore, the beneficial effects of the topical application of the two extracts were demonstrated with an increase in hydration and an improvement in skin barrier function, which were more pronounced for the SEL. The high-order lipidic structure and orthorhombic package determined by ATR-FTIR also supports the increase of the barrier function in a way similar to that of the stratum corneum (SC) lipid organization [23]. However, the acidic pH of the WEL, which is similar to that of the stratum corneum, seems to be very important for the emulation of skin permeability. Therefore, it can be concluded that WE lanolin is the most appropriate to produce artificial membranes due to its pH value, transepidermal water loss, and ability to reproduce the results of penetration of actives through the skin.

## 4. Conclusions

New approaches to reproduce skin absorption, such as the creation of artificial skin substitutes, are being investigated. Given the close similarity between human SC lipids and lanolin in their composition and structure, new artificial skin models composed of different wool waxes have been developed. This work showed that the new lanolin-based artificial membranes have the potential to be used as screening models to determine the permeability of actives intended to be administered topically.

Two types of lanolin were examined, one extracted in an aqueous medium with surfactants (WE) and the other with organic solvents (SE). The pH value of the lanolin provided information on the percentage of ionization of the APIs in the vicinity of the membranes, thus giving information on the capacity of the APIs to pass through the membranes. The pH values of the membranes were around 6.5 for the SEL and 5.5 for the WEL membranes, in this case, similar to the pH of the skin surface. In addition, the similar TEWL values for lanolin membranes compared to skin indicated that the lanolin-induced barrier in these membranes was close to the skin barrier.

To determine the pros and cons of lanolin-based synthetic membranes as models of mammalian skin, both skin permeation and skin penetration studies were performed with two active compounds. Diclofenac sodium and lidocaine were selected because they have different chemical natures (acidic and basic, respectively) and different lipophilicities. Lidocaine is the more lipophilic compound (at neutral pH) with a smaller molecular weight; therefore, it has the highest log K_o/w_, which indicates theoretical greater permeability. In the permeation assay with SEL, a better correlation was obtained with the less permeable compound diclofenac sodium. This assay suggested the feasibility of using artificial membranes with SE lanolin as a model for percutaneous absorption studies, even though the lipophilic barrier should be improved. The penetration profiles of the APIs through the SEL and WEL membranes were determined. The two membranes diminished penetration and could be considered good membrane surrogates for skin permeability studies. However, the WEL membranes, with a pH value similar to that of the skin surface, promoted the higher degree of permeability diminution of the two drugs, similar to that found for the skin.

Further experiments should be conducted to obtain better reproduction between the same type of membrane. The membrane-lipids assembly procedure should be optimized to increase lipid binding. In addition, the pool of tested compounds should be expanded, especially the number of highly lipophilic compounds. Broader ranks of molecular weights and pKa need to be fully explored. Lanolin membranes present the advantage of providing permeation and penetration profiles similar to those using skin. Thus, this feature could be helpful in the discrimination of APIs or topically administered formulation systems. In the early stages of cosmetic or pharmaceutical product developments, different formulations and vehicles could be tested with these artificial lanolin-based membranes.

## Figures and Tables

**Table 1:** HPLC analytical conditions and parameters for diclofenac sodium (DS) and lidocaine (LIDO).

Parameters	Diclofenac Sodium	Lidocaine
Extractor solvent	CH_3_CN (+CF_3_COOH 0.5%)	CH_3_OH
Column	LiChrocart^®^ 250-4 Lichrosphere^®^ 100RP-18, 5 µm	LiChrocart^®^ 125-4 Lichrosphere^®^ 100RP-18, 5 µm
Wavelength (nm)	254	205
Injection volume (µL)	20	20
Mobil phase (flux)	66% CH_3_OH 34% H_3_PO_4_ 0.7% (1 mL/min)	70% NaH_2_PO_4_, 0.05 M pH 7.4 30% CH_3_CN (1 mL/min)
Linear regression Equation (R^2^)	*A* = 80,050(*DS*) − 2484 (0.9997)	*A* = 414,046(*LIDO*) − 68,532 (0.9999)
LoD/LoQ (µg/mL)	0.07/0.22	0.12/0.35
Precision (%CV) Intra day	2.05 ± 0.71	5.21 ± 2.86
Inter day	6.02 ± 1.98	5.30 ± 3.03

**Table 2:** Amount of lanoline in lanolin-based membranes, transepidermal water loss (TEWL), and pH of all membranes.

Membranes	Lanolin Amount (mg)	pH	TEWL (g/hm^2^)
Skin	-	5.7 ± 0.3	5.9 ± 2.3
NP	-	6.5 ± 0.2	52.5 ± 9.0 *
NP-SEL 5%	14.3 ± 1.8	6.6 ± 0.3	14.9 ± 3.6
NP-WEL 5%	13.3 ± 3.8	5.3 ± 0.2	15.2 ± 1.2

* accounts for significant difference related to the skin, *p* < 0.05.

**Table 3:** Octanol-water distribution coefficients (log D), pKa values, and molecular weights (MWs) were obtained from the ChemAxon platform and log Kp in silico permeability (Potts and Guy).

Compound	pKa	LogD at pH 5.5	LogD at pH 7.4	MW	LogKp (Potts & Guy)
Diclofenac Sodium	4.15 [23]	2.75	1.10	318.1	−7.42
Lidocaine	7.70 [24]	0.61	2.33	234.3	−5.99

**Table 4:** Average values of Kp, J, Cmax, and area under the curve (AUC) of penetration of the DS 3% and LIDO 2% solution for all membranes.

Membrane	API	Kinetics of Release	J(µg/cm^2^/t) ^a^	Kp (cm/t)	C max (µg/mL)	AUC (a.u.)
SKIN	DS	Ord 0	3.94	0.0001	38.10	167.55
	LIDO	Ord 0	2.29	0.000125	49.95	109.30
NP	DS	Higuchi	2307.12 *	0.0642 *	3056.54 *	14,821.56 *
	LIDO	Higuchi	1360.27 *	0.0843 *	2021.17 *	13,197.15 *
SEL	DS	Ord 0	30.40 *	0.00121 *	615.29 *	1977.11 *
	LIDO	Higuchi	343.05 *	0.0213 *	1540.43 *	7341.61 *
WEL	DS	Ord 0	6.18	0.000208	48.43	317.06
	LIDO	Ord 0	4.85	0.000269	144.78	509.19

* stands for significant differences, *p* < 0.05, compared to the skin membrane for DS and LIDO, respectively. ^a^ Flux units: Higuchi model (µg/cm^2^/h^½^) and Ord 0 (µg/cm^2^/h).

**Table 5:** The pKa, log Kp in silico, log kp in vitro from the experimental kinetic assay.

Compound	pKa	Log Kp	Log Kp		
		In Silico	Permeation Infinite Dosage		
		(Potts & Guy)	Skin	SEL	WEL
Diclofenac Sodium	4.15	−7.42	−3.98	−2.92	−3.68
Lidocaine	7.70	−5.99	−3.90	−1.67	−3.57

## Data Availability

Not Applicable.
